# Acceptable Weight Ranges for Research Tissue Procurement and Biorepositories, 2015–2017

**DOI:** 10.1089/bio.2018.0068

**Published:** 2018-12-17

**Authors:** David G. Nohle, Randal L. Mandt, Marta E. Couce, Anil V. Parwani, Leona W. Ayers

**Affiliations:** ^1^ Ohio Consortium, Cooperative Human Tissue Network (CHTN) Midwestern Division, Columbus, Ohio.; ^2^ Department of Pathology, The Ohio State University, Columbus, Ohio.; ^3^ Department of Pathology, Case Western Reserve University, Cleveland, Ohio.

**Keywords:** research tissue weight, biorepository, anatomic site weight groups

## Abstract

***Background:*** The Cooperative Human Tissue Network, Midwestern Division, is a National Cancer Institute-funded program that provides quality research biospecimens to qualified investigators. Consented human tissues are procured according to researcher specifications for weight (size) and preservation type; weights of samples in significant demand and limited supply are negotiated. Weights of procured tissues are entered into a dedicated biospecimen database. This study seeks to provide guidance for acceptable tissue weights for researchers.

***Methods:*** Tissue weights by year and anatomic site were retrieved from the database for primary malignant tissues. The total number of tissues included was 5141. Statistical evaluation of data included the number of tissues for each year, anatomic site as well as minimum, maximum, average weights, standard deviation, and standard error. Anatomic sites with few tissues were excluded.

***Results:*** “Stock price” type graphs were constructed to show an average as “volume” with both full weight ranges and range that accommodated 80% of tissues. Average weight and number of sample trends varied by anatomic site. Tissues fell into four weight groups; 10 and 90 percentile boundaries were calculated for each. Smallest average research tissue weights for middle 80% were recorded for prostate and oropharynx (140 mg). Second weight group included tonsil, thyroid, breast, oral cavity, larynx, pancreas, salivary gland, skin, tongue, lung, and parotid (265 mg). The third group included stomach, cervix, colon, esophagus, endometrium, bone, brain, bladder, small bowel, uterus, liver, kidney lymph node, adrenal, and ovary (513 mg). The fourth and heaviest weight group included soft tissue tumors and spleen (1201 mg).

***Conclusions:*** Since tissue weights are not usually included in recommendations for research tissue procurement or for frozen tissues stored in biorepositories, we offer this data as a practical guide to researcher acceptable tissue weights for selected sites based on a 3-year researcher request and acceptance history.

## Introduction

The Midwestern Division (MWD) of the Cooperative Human Tissue Network (CHTN) is a National Cancer Institute (NCI/NIH)-funded resource to provide research biospecimens to qualified investigators.^1^ Six CHTN divisions serve the investigators in the United States and Canada.^2^ MWD serves north central U.S. states and Canada but is in consortium with the other CHTN divisions to supply researcher's needs nationwide.

Donor-consented tissue samples are procured from MWD Ohio Consortium institutions according to researcher specifications for size (weight) and preservation type with the exception of those samples in significant demand and limited supply may have negotiated shipped weights. The investigator is responsible for calculating their tissue needs based on the testing anticipated. Guidance on sample weights for research procurement and biorepositories is not currently available in published literature^3–5^ or best practices.^6,7^

## Methods

Procured research tissue weights and preservation method were specified by approved CHTN investigators. Weights of procured tissues were measured using an OHAUS scale, model no. SPX222 (OHAUS Corporation, Parsippany, NJ) that has a capacity of up to 220 g and a readability of 0.01 g with a pan size of 4.7 inches. Procured sample weights were entered into CHTN MWD software (Research Tissue Procurement Information System, RTP-IS). Weighed samples were immediately preserved according to investigator's request.

Formalin-fixed samples were most often requested (42%), followed by frozen (32%), and then fresh (26%). Fresh samples were collected in investigator provided media (59.6% of fixed and 15.8% of all) followed by CHTN media (39.0% and 10.3%).^8^

For each investigator sample procured, an adjacent tissue quality control (QC) sample was processed in paraffin and subsequently a hematoxylin- and eosin-stained tissue section was evaluated. This adjacent sample tissue was examined by a pathologist for appropriate cell morphology, cellularity, and necrosis.^9^ Samples that met criteria of >20% requested morphology and <80% necrosis were shipped. Most tissue samples had 60%–95% requested material and little or no necrosis. The percentage of tissues passing QC is monitored by the Anatomic Pathology Quality Improvement program. Tissue QC acceptance ranges were from 92.8% to 98.2% monthly.

Shipped tissue weights were mined from the RTP-IS database for years 2015–2017.^10^ Samples by year and anatomic site for only primary malignant tissue samples with weight recorded (vs. size dimensions or liquid volumes) were included. The number of samples (*N*) evaluated was 2119 in 2015, 1464 in 2016, and 1558 in 2017 for a total of 5141 sample weights. Statistics included number of samples for each year and anatomic site as well as minimum, maximum, average, standard deviation, and standard error. Anatomic sites with too few samples were excluded.

A “stock price” type graph was constructed to show an average as “volume” with both full weight ranges and range that accommodated 80% of samples. A review of this graph prompted us to combine years for each site and sort by averages to identify weight groups in a second graph. A third graph was constructed to display data about the resulting groups.

## Results

**FIG. 1.**
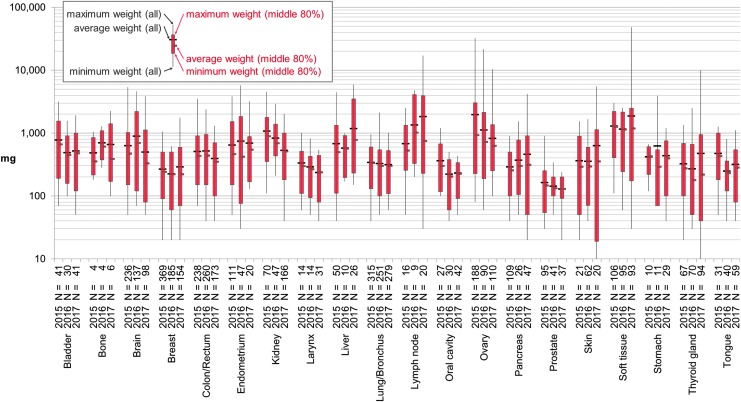
Shipped sample weight for 20 anatomic sites by year 2015–2017. Shipped tissue weights are recorded in the Research Tissue Procurement Information System for all procured tissues. The *top* and *bottom* 10 percentiles (%) were calculated so that the *middle* 80% could represent each combination of anatomic site and year without undue influence from less common outliers. The full range of weights for all samples from each anatomic site and year is shown with the corresponding average (*thin black line* and *black tick mark*) as well as the minimum, maximum, and average of the *middle* 80% (*thick red bar* and *smaller red tick mark*). A log scale is used to simultaneously display smaller weight details, whereas showing the larger weights for context. *N*, number of samples.

The number of tissue samples and average weight by anatomic site included flat, rising, and falling trends and are shown in Figure 1. Each anatomic site had its own requested weight and weight distribution pattern.

**FIG. 2.**
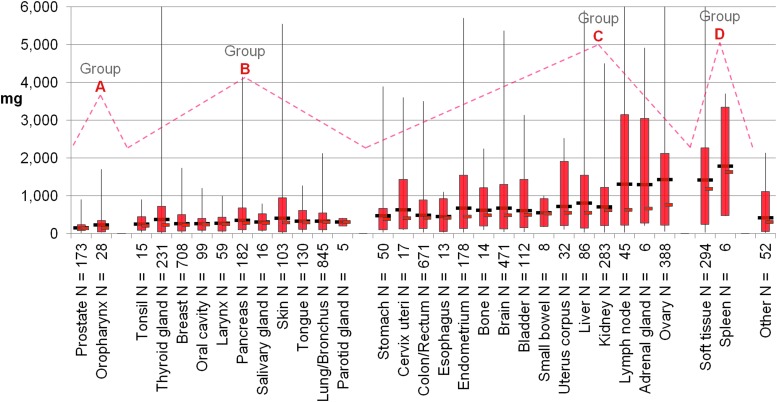
Shipped sample weight for 30 anatomic sites combining 2015–2017 for each. Graphed as in Figure 1 except that a linear scale is used (cropping four sites with weights >10,000 mg) and shows how less common sample weights in the *top* 10 percentiles would skew an average that included them. Four groups **(A–D)** were identified by eye based on average weight of the *middle* 80 percentiles. Eighteen other anatomic sites had few samples and are grouped as *other*. *N*, number of samples.

Tissue weights fell into four groups; 10 and 90 percentile boundaries were calculated for each (Fig. 2). Smallest average research tissue weights for middle 80% were recorded for prostate and oropharynx (140 mg). Second weight group included tonsil, thyroid, breast, oral cavity, larynx, pancreas, salivary gland, skin, tongue, lung, and parotid (265 mg). Third group included stomach, cervix, colon, esophagus, endometrium, bone, brain, bladder, small bowel, uterus, liver, kidney lymph node, adrenal, and ovary (513 mg). The fourth and largest weight group included soft tissue and spleen at average distributed weight of 1201 mg.

## Discussion

Tissue weights for the same anatomic site have a range that is similar over the 3-year study period with only minor change in weights requested/distributed. The 3-year trend displayed for each anatomic site could indicate whether research testing is increasing or available tumor/sample size is decreasing. The anatomic site difference in target cells, nuclear size, and stromal density in different organ tissue types and the type of testing performed by investigators likely contribute to the differences between tissue weights by tissue type. The size of the submitted tissue also influences the amount of tissue procured per sample. Neither the procurement agents nor the investigators were regularly coached in their weight requests.

**FIG. 3.**
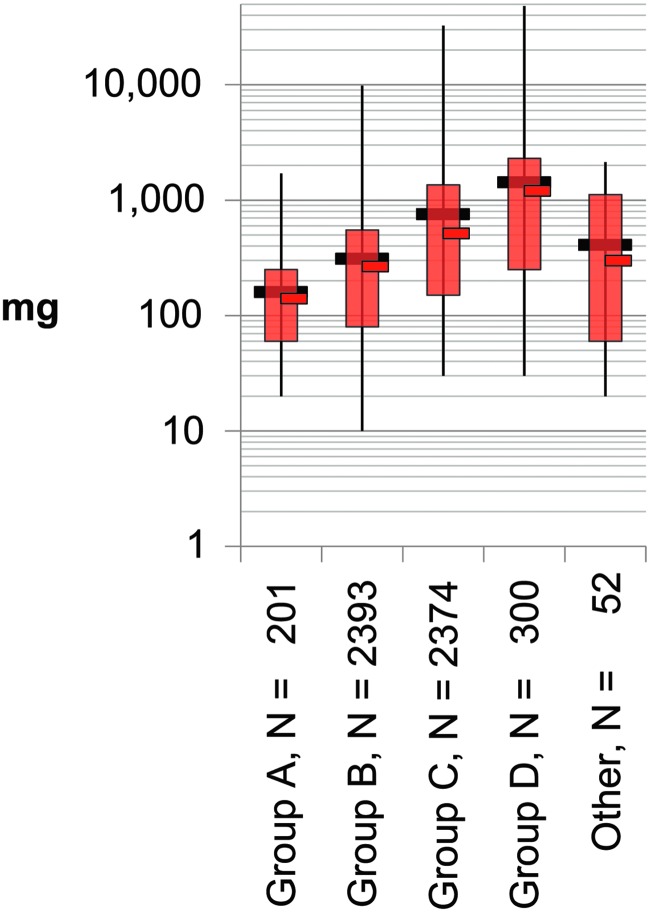
Weight groups of anatomic sites for shipped samples, 2015–2017. Eighteen anatomic sites were identified as four groups with few samples of low-incident tissues grouped as *other*. Each group was combined and graphed as in Figure 2 but on log scale. Recommendations for weight or size are not provided by tissue type/site, so a single size may be stored by a biorepository. *N*, number of samples.

The results show that for all the likely variations in what experienced investigators were studying, the amount of quality-specified tissue (investigators receive a QC report) is similar for the same organ systems over a 3-year period. Interestingly, the major change that occurred in the investigator request was for more fresh tissue stabilized in the investigator's own liquid media compared with the previous 8 years (8.73%–21.06%).^8^ The four weight groups identified provide guidance for desired weight sizes (Fig. 3; Table 1).

**Table 1.** Shipped Sample Weight Statistics for Groups of Anatomic Sites for 2015–2017

*Anatomic site group*	N	*Minimum weight (mg)*	*10th percentile*	*Middle 80% average weight (mg)*	*All 100% average weight (mg)*	*90th percentile*	*Maximum weight (mg)*	*Standard deviation*	*Standard error*
Group A	201	20	60	139.9	158.9	250	1700	146.8	10.4
Group B	2393	10	80	265.1	309.1	550	9850	344.9	7.1
Group C	2374	30	150	513.3	753.4	1357	32,630	1476.6	30.3
Group D	300	30	250	1201.4	1420.4	2300	48,000	2878.2	166.5
Other	52	20	60	297.3	408.1	1116	2140	469.2	65.7

We conclude after study of tissue weights commonly accepted by researchers that there may be advantage to frozen storage of at least four different sizes. Frozen storage of minimal weights for highly sought after tissues such as prostate may allow service to more investigators. Storage of larger weights may offer some advantage to study of soft tissue sarcomas, for example. Standard sample size requirements for biorepositories may depend on the anatomic site(s) that are to be included (as well as the intended purpose of the collection).

Extensive data on investigator-acceptable weights can serve as a practical guide for research tissue procurement as such data do not appear in the available published literature. Acceptable tissue weights that are investigator defined also support decisions for biorepository frozen tissue storage weights. Highly requested anatomical tumor sites such as prostate and breast could be stored as individual aliquots at lower individual weights to provide tissue access to more investigators and to avoid detrimental freeze–thaw cycles.

Since tissue weights are not usually included in published recommendations for frozen tissues stored in biorepositories, we offer this data as an initial reference guide to both issue procurement services and biorepositories as researcher-acceptable tissue weights for selected anatomical sites of quality-controlled research tissue samples. Guidance is based on 3 years of CHTN MWD researcher sample weight request and acceptance history. We anticipate using this weight data to quality check investigator tissue weight requests in the future. Others could find this data useful for doing similar weight/size checks for excessive or insufficient tissue requests by investigators. There will likely be outliers based on unusual testing requirements allowing provider inquiry to justify more or less tissue if available.
